# Clinical Phenotype in Individuals With Birk-Landau-Perez Syndrome Associated With Biallelic *SLC30A9* Pathogenic Variants

**DOI:** 10.1212/WNL.0000000000207241

**Published:** 2023-05-23

**Authors:** Dora Batia Dyne Steel, Federica Rachele Danti, Mohamed Abunada, Benjamin Kamien, Sony Malhotra, Maya Topf, Marios Kaliakatsos, Jane Valentine, Andrea Hilary Nemeth, Sandeep Jayawant, Kimberley M. Reid, Kshitij Mankad, Sniya Sudhakar, Hilla Ben-Pazi, Katy Barwick, Manju A. Kurian

**Affiliations:** From the Department of Developmental Neurosciences (D.B.D.S., K.M.R., M.A.K.), UCL Great Ormond Street Institute of Child Health; Departments of Neurology (D.B.D.S., F.R.D., M.K., K.B., M.A.K.) and Radiology (K.M., S.S.), Great Ormond Street Hospital, London, United Kingdom; Pediatric Neurology Department (M.A.), al-Rantisi Pediatric Hospital, Gaza; Department of Paediatrics (B.K., J.V.), University of Western Australia Medical School, Perth, Australia; Scientific Computing Department (S.M.), Science and Technology Facilities Council, Didcot, United Kingdom; Centre for Structural Systems Biology (M.T.), Leibniz-Institut für Experimentelle Virologie and Universitätsklinikum Hamburg-Eppendorf (UKE), Germany; Nuffield Department of Clinical Neurosciences (A.H.N.), University of Oxford; Department of Paediatric Neurology (S.J.), Oxford Radcliffe Hospitals; and Neuropediatric Unit (H.B.-P.), Shaare Zedek Medical Centre, Jerusalem, Israel.

## Abstract

**Background and Objectives:**

Birk-Landau-Perez syndrome is a genetic disorder caused by biallelic pathogenic variants in *SLC30A9* presenting with a complex movement disorder, developmental regression, oculomotor abnormalities, and renal impairment. It has previously been reported in 2 families. We describe the clinical phenotype of 8 further individuals from 4 unrelated families with *SLC30A9*-related disease.

**Method:**

Following detailed clinical phenotyping, 1 family underwent research whole-genome sequencing (WGS), 1 research whole-exome sequencing, and 2 diagnostic WGS. Variants of interest were assessed for pathogenicity using in silico prediction tools, homology modeling, and, where relevant, sequencing of complementary DNA (cDNA) for splicing effect.

**Results:**

In 2 unrelated families of Pakistani origin (1 consanguineous and 1 not), the same homozygous missense variant in *SLC30A9* (c.1253G>T, p.Gly418Val) was identified. Family 1 included 2 affected brothers, and family 2 one affected boy. In family 3, also consanguineous, there were 4 affected siblings homozygous for the variant c.1049delCAG, pAla350del. The fourth family was nonconsanguineous: the 1 affected individual was compound heterozygous for c.1083dup, p.Val362Cysfs*5, and c.1413A>G, p.Ser471=. Despite phenotypic variability between the 4 families, all affected patients manifested with a progressive hyperkinetic movement disorder, associated with oculomotor apraxia and ptosis. None had evidence of severe renal impairment. For the novel missense variant, the conformation of the loop domain and packing of transmembrane helices are likely to be disrupted based on structure modeling. Its presence in 2 unrelated Pakistani families suggests a possible founder variant. For the synonymous variant p.Ser471=, an effect on splicing was confirmed through cDNA analysis.

**Discussion:**

Pathogenic variants in *SLC30A9* cause a progressive autosomal recessive neurologic syndrome associated with a complex hyperkinetic movement disorder. Our report highlights the expanding disease phenotype, which can present with a wider spectrum of severity than has previously been recognized.

*SLC30A9* encodes a cation transporter thought to be primarily involved in cellular zinc homeostasis, designated ZnT-9.^1^ Recently, a novel syndrome consisting of a movement disorder, neurodevelopmental regression, oculomotor apraxia, and progressive renal impairment was described in a single large Bedouin kindred with a homozygous *SLC30A9* in-frame deletion.^2^ A single individual from a second family with compound heterozygous variants has also very recently been reported.^3^ This condition has been designated Birk-Landau-Perez syndrome (BLPS).^4^

We describe 4 further families with different biallelic *SLC30A9* variants associated with this distinctive clinical syndrome and prominent progressive hyperkinetic movement disorder. This provides confirmation of the significance of biallelic *SLC30A9* variants in neurologic disease, as well as expanding our understanding of the phenotype of this newly described disorder.

## Methods

### Standard Protocol Approvals, Registrations, and Patient Consents

For those participants who underwent genetic testing on a research basis, ethics approval was given by the Bloomsbury Research Ethics Committee (REC: 13LO168). Written informed consent for participation in the study was obtained from all participants or their guardians. Authorization has been obtained for the disclosure of recognizable people shown in videos.

### Participant Recruitment

Families with children affected by etiologically undiagnosed movement disorders were recruited for molecular genetic investigations. Detailed clinical phenotyping was undertaken by a pediatric neurologist or clinical geneticist. For family 1, whole-genome sequencing (WGS) was undertaken on 1 affected member, with subsequent segregation studies by Sanger sequencing on both parents and the other affected sibling. Family 3, recruited to the research program several years earlier, underwent autozygosity mapping on both parents, 4 affected children, and 1 unaffected child. Two affected family members then underwent whole-exome sequencing (WES), and segregation of variants was subsequently confirmed by Sanger sequencing. Families 2 and 4 were referred for diagnostic testing by their clinical teams.

### Molecular Genetic Investigations

Lymphocytic DNA for all families was extracted from peripheral blood. For family 1, WGS was undertaken on a triome basis for 1 affected sibling (F1(II-2)) and both parents. WGS was performed by BGI using the DNBSeq next-generation sequencing technology platform, using 100 base pair (bp) paired-end reads. Variant calling was performed using SAMtools,^5^ SOAPsnp,^6^ and Genome Analysis Toolkit.^7^ Reads were aligned to the GRCh37/hg19 reference genome. Variant prioritization was performed using QIAGEN Ingenuity Variant Analysis followed by variant analysis using Alamut Visual 2.11.

A shortlist of rare homozygous variants (minor allele frequency <1%; no known homozygotes in the Genome Aggregation Database^8^ population database) in a large customized virtual panel of 3,477 genes associated with movement disorders and related conditions was compiled. In silico tools used to predict variant pathogenicity included Combined Annotation Dependent Depletion, Sorting Intolerant From Tolerant,^10^ PolyPhen2,^11^ MutationTaster (v.2013),^12^ and PROVEAN.^13^

Family 2 underwent diagnostic WGS through the National Health Service (NHS) Genomic Medicine Service. Only the proband and mother participated.

For family 3, WES and single nucleotide variation (SNV [formerly SNP]) array were performed at UCL Genomics. SNV array used Illumina Infinium HD Assay Ultra Protocol with a combination of the manual and automated methods and was performed for all 4 affected siblings. WES used the Nonacus Cell3 Target kit and protocol. Shared regions of homozygosity were then examined for potentially pathogenic variants in relevant genes.

For family 4, diagnostic triome WGS was performed by Genome.One, a commercial organization, using the TruSeq Nano DNA Library Preparation Kit and Illumina 150 bp paired-end sequencing to a mean depth of 30×. Reads were aligned to the GRCh37/hg19 reference genome, and variant calling used Genome Analysis Toolkit and ClinSV.^14^ Variant prioritization followed a proprietary algorithm.

Confirmation of variants and segregation analysis was undertaken for families 1 and 3 by Sanger sequencing as previously described.^15^ For family 2 and family 4, the variants were called with high confidence according to the prespecified criteria of the respective sequencing institutions (for Genome.One, this includes coverage depth >35×, genotype quality ≥99.0, and mapping quality ≥60), so Sanger confirmation was not required.

### Structure-Function Homology Modeling

The sequence of human zinc transporter 9 SLC30A9 from UniProt^16^ (Q6PML9) was used to identify a suitable structural template by sensitive sequence search using the HMMER program^17,18^ (at E value 0.001). The structure of a zinc transporter from a cation diffusion facilitator family (*YiiP*), recently derived by cryo-electron microscopy was used as the best template structure to model human SLC30A9 (26% sequence identity) (Protein Data Bank ID: 5VRF). Because of the low sequence identity with the template, we searched for a structural model for this protein in the AlphaFold Protein Structure database.^19,20^ The AlphaFold model for the stretch of residues 221–449 harboring the variant of interest G418 was selected for further analysis. The multiple sequence alignment was generated using the ConSurf server^21^ with the following parameters: method of sequence search—HMMER, HMMER E value = 0.0001; database used—UNIREF90; number of HMMER iterations—3; maximum percent identity between sequences—95%; and minimum percent identity between sequences—35%. The nonsynonymous substitution was mapped onto the structure using the swapaa command in UCSF Chimera^22^ based on the Dunbrack backbone-dependent rotamer library^23^ and taking into account the lowest clash score, highest number of H-bonds, and highest rotamer probability to investigate their structural and functional effects. The Positioning of Proteins in Membranes server^24^ was used to calculate the position of the membrane for the modeled structure. TMHHM was used to predict the position of the variant relative to transmembrane helices.^25^

### Splicing Assay

To assess the effect of the putative splicing variant in proband 4, analysis of complementary DNA (cDNA) was undertaken. RNA was extracted from pelleted patient-derived fibroblasts using the Qiagen RNeasy Mini Kit according to the manufacturer's instructions. Reverse transcription to generate cDNA from the extracted RNA was then performed using the Invitrogen SuperScript III Reverse Transcriptase kit. Primers were designed to cover the relevant splice site with the following sequences:

Forward: GCATGGTCTCAGCATTCCTC
Reverse: TTCTCAAGTCTATCTACTTCAGCTCCT

Following primer optimization, a touchdown PCR protocol with a 30-second extension time and 64°C annealing temperature was chosen. PCR was performed using the cDNA. The product then underwent gel electrophoresis on a 3% agarose gel stained with SYBR Safe for 30 minutes at 100 V, and images were obtained using a ChemiDoc Imager, with amplicon size assessed using the GeneRuler 100 bp DNA Ladder from ThermoFisher Scientific. The electrophoretic bands were manually excised under direct vision with an ultraviolet lamp. A sequencing reaction was then performed on the cDNA from each band using the BigDye Terminator v1.1 Cycle Sequencing Kit from Thermo Fisher Scientific before sequencing by the Great Ormond Street diagnostic genetics laboratory on an ABI 3730xl DNA Analyzer. Sequencing results were analyzed using FinchTV.

### Data Availability

Due to the terms of our ethical approval, we are not permitted to share participants' genomic or exomic data. The authors can be contacted with any queries about the data or their analysis.

## Results

### Clinical Description

**Figure 1:**
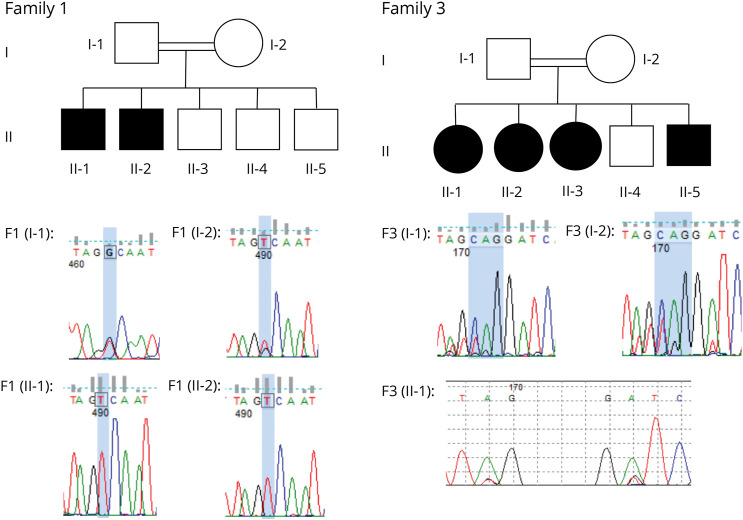
Pedigrees and Sequencing Data Above: pedigrees of families 1 and 2. Key: circle: female; square: male; double horizontal line: consanguineous union; filled shape: affected individual. Below: chromatograms showing Sanger sequencing results for selected family members. Family 1: affected base is shaded in blue; note that parents are heterozygous (double trace) and affected children are homozygous. Family 2: the affected codon in parental traces is shaded in blue; note double trace emerging (to the left) indicating heterozygosity. Representative trace from 1 affected child's shows codon homozygously absent.

Pedigrees for the multiplex families (1 and 3) are shown in Figure 1. Family 1 is a British family of Pakistani origin, in which the parents were first cousins. The 2 eldest brothers, F1(II-1) and F1(II-2), presented with a similar neurologic phenotype. Both boys were born at term with unexplained low birth weight, <0.4th centile. Both achieved early neurodevelopmental milestones at the later end of the normal range, walking at 18 months, and both were diagnosed with moderate learning difficulties but were able to continue in mainstream education with specialized support.

Patient F1(II-2) presented with a movement disorder at around age 7 years. Patient F1(II-1) did not develop a movement disorder until age 16 years, and it remains somewhat milder than his brother's. In both cases, the movement disorder consists of continuous, irregular, low-amplitude choreiform movements and low-amplitude myoclonic jerks of all 4 extremities, together with some subtle perioral dyskinesia and mild dystonic posturing on walking (Videos 1–3). Gait was effortful for both, and by age 16 years, patient F1(II-2) was only able to walk a few steps without assistance. Patient F1(II-2), but not F1(II-1), developed a progressive thoracic kyphosis. Neither of the boys displayed any spasticity, focal weakness, or any convincing signs of cerebellar involvement, although speech was moderately dysarthric in both. Both also had musculoskeletal contractures: in F1(II-1), these involved the fingers bilaterally, and in F1(II-2), the elbows and the left foot. Patient F1(II-1) had mild difficulties with saccadic eye movements, and F1(II-2) developed clear oculomotor apraxia. Both had subtle bilateral ptosis. Patient F1(II-1) also has moderate bilateral sensorineural hearing impairment. Neither patient had any impairment of kidney function at the time of assessment, but patient F1(II-2) was known to pediatric nephrology for unexplained small echogenic kidneys, and also under investigation for possible hypertension.

10.1212/207241_Video_1 Video 1 Upper limb movements in patient F1(II-1): patient F1(II-1) walking: note bilateral upper limb posturing. He is then asked to extend both arms, bringing out distal choreiform movements.Download Supplementary Video 1 via http://dx.doi.org/10.1212/207241_Video_1

10.1212/207241_Video_2 Video 2 Gait in patient F1(II-2): patient F1(II-2) walking: note generalized dystonia including a degree of anterocollis and fixed contracture of the left Achilles tendon.Download Supplementary Video 2 via http://dx.doi.org/10.1212/207241_Video_2

10.1212/207241_Video_3 Video 3 Upper limb movements in patient F1(II-2): patient F1(II-2) is asked to extend both arms, bringing out both dystonic posturing and choreiform movements. He is then asked to open and close his fists, eliciting more dystonia and possible amplitude reduction.Download Supplementary Video 3 via http://dx.doi.org/10.1212/207241_Video_3

**Figure 2:**
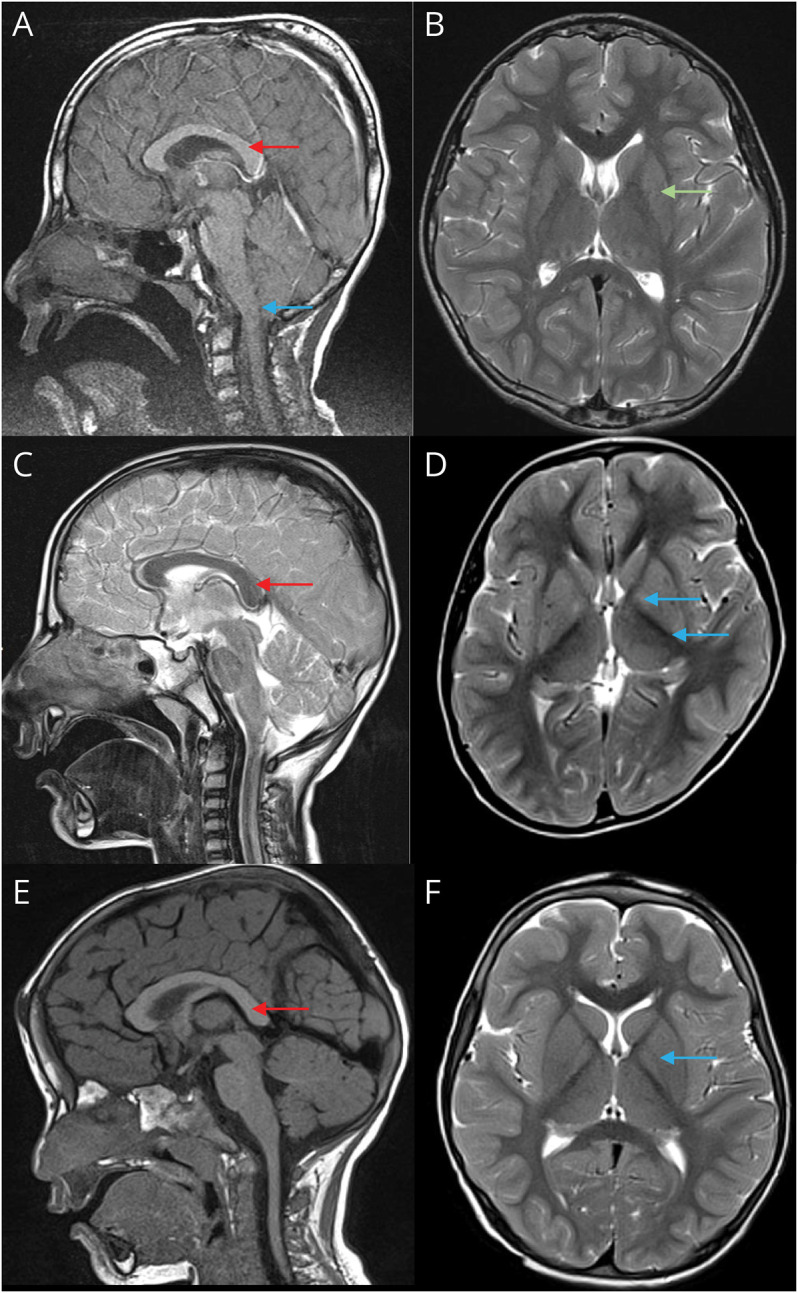
MRIs From Participants (A) F1(II-2) sagittal, T1 weighted, showing dysmorphic corpus callosum (red arrow) and shallow pontomedullary sulcus (blue arrow). (B) F1(II-2): axial, T2 weighted, showing small-volume basal ganglia (green arrow). (C) F2 sagittal, T2-weighted, showing vertical posterior corpus callosum (red arrow). (D) F2 axial, T2 weighted, showing symmetrical hyperintensity of the globus pallidus and posterior putamen (blue arrows). (E) F3(II-5) sagittal, T1-weighted, showing vertical posterior corpus callosum (red arrow). (F) F3(II-5) axial, T2-weighted, showing small globus pallidus with altered signal intensity (blue arrow).

Brain MRI for patient F1(II-2), aged 11 years, was locally reported as normal, but review by a pediatric neuroradiologist identified subtle abnormalities including a shallow pontomedullary sulcus, short clivus with retroslanting odontoid, low cerebellar tonsils, a mildly dysmorphic corpus callosum, and borderline small volume putamen and caudate (Figure 2). Detailed diagnostic metabolic testing was nondiagnostic including normal urea and electrolytes, calcium, magnesium, liver function tests, very-long-chain fatty acids, ammonia, creatine phosphokinase, transferrin glycoforms, biotinidase, plasma amino acids, urine organic acids, glycosaminoglycans, and urine guanidinoacetate. He was found to have autoimmune hypothyroidism treated with levothyroxine. CSF showed normal paired glucose, lactate, glycine, and pterins, with a mild isolated reduction in 5-hydroxyindoleacetic acid (51 nmol/L, range 58–220), which was not thought to be significant. A muscle biopsy showed mitochondrial DNA at borderline levels of 40%, but respiratory chain enzyme levels and sequencing of the mitochondrial genome were normal. Serum levels of zinc, copper, and selenium were normal. Both brothers were treated with a low dose of levetiracetam, which they felt helped reduce their abnormal movements.

Family 2 is also British Pakistani. The parents are not consanguineous but are from the same geographic area. F2 has 2 healthy older siblings and was born by induced labor at 38 weeks due to intrauterine growth restriction, with a low birth weight of 2.1 kg. He walked at 14 months, but from age 2 years, his gait became unsteady, and he started to toe-walk. Developmental milestones were slightly delayed in all areas relative to his siblings. On examination, at age 4 years and 8 months, dystonia and low-amplitude myoclonic jerks were evident in all limbs, and orolingual dyskinesia was seen (Video 4). On purposeful movements such as finger-nose pointing and finger opposition, he was bradykinetic. Mild spasticity and brisk deep tendon reflexes were present in the lower but not upper limbs, and Achilles tendons were tight. Plantars were downgoing. He was microcephalic with a *Z* score of −4.08 for head circumference and had very subtle ptosis on the right only. He had difficulties with both saccadic and smooth pursuit eye movements. Moderate bilateral sensorineural hearing loss was diagnosed at age 5 years.

10.1212/207241_Video_4 Video 4 Movement disorder in patient F2: patient F2 is sitting: he is asked to extend his arms, and distal choreiform movement and low-amplitude myoclonus is seen. During finger-nose pointing, as well as inaccuracy bradykinesia is notable. He is then shown walking, demonstrating crouched gait, unsteadiness, toe-walking, and dystonic upper limb posturing.Download Supplementary Video 4 via http://dx.doi.org/10.1212/207241_Video_4

Brain MRI showed very subtle symmetrical T2 hyperintensity of the globus pallidus and posterior putamen, minimal cerebellar volume loss, and a dysmorphic (posteriorly vertical) corpus callosum (Figure 2). Microarray and initial neurometabolic testing were normal, except that both urea and creatinine were modestly elevated: at age 5 years, he was found to have moderately impaired renal function (estimated glomerular filtration rate of 40 mL/min/1.72 m^2^ [range >90 mL/min/1.72 m^2^]) with small echogenic kidneys and no proteinuria.

Family 3 is a Palestinian family of Egyptian origin with multilevel consanguinity: the parents are double first cousins. They are not known to have Bedouin heritage. The 4 affected siblings all presented within the first 2 years of life with a progressive movement disorder. Patient F3(II-1) never walked, but the other 3 (F3(II-2), F3(II-3), and F3(II-5)) were able to walk briefly from about 20 months old, losing ambulation at 2 years. All 4 children had evidence of bradykinetic voluntary movements, with generalized dyskinesia and dystonia involving the limb extremities and trunk. Ptosis and oculomotor apraxia were also universally evident. All 4 had a severe intellectual disability with limited verbal communication. None are known to have any renal impairment.

Brain MRI for patient F3(II-5) was reported locally as normal, but pediatric neuroradiologic review again identified subtle abnormalities: minimal cerebellar volume loss; the globus pallidus was small with altered signal intensity; the corpus callosum showed posterior vertical morphology; and there was unusual perirolandic sulcation on the right (Figure 2). Due to resource constraints, neither imaging of the other siblings nor serum zinc testing could be performed.

Family 4 is White Australian, and the parents are not consanguineous. The proband was diagnosed with bilateral sensorineural hearing loss at age 13 months and managed with cochlear implants. She had global developmental delay and a mildly abnormal gait but did acquire the ability to walk and run. From age 5 years, she developed a slowly progressive movement disorder involving her trunk, limbs, face, and eyes. Examination at age 10 years showed generalized dystonia, ataxia, bilateral ptosis, and oculomotor apraxia (Videos 5 and 6). Brain MRI (aged 2 years) was normal. Kidney function and renal ultrasound at age 10 years were normal. Serum zinc and manganese levels were normal.

10.1212/207241_Video_5 Video 5 Movement disorder in patient F4: patient F4 is seen walking with assistance, showing four-limb dystonic posturing. She is asked to open and close her hands, which she does with difficulty.Download Supplementary Video 5 via http://dx.doi.org/10.1212/207241_Video_5

10.1212/207241_Video_6 Video 6 Oculomotor apraxia in patient F4: patient F4 demonstrates oculomotor apraxia: in clip 1, she moves her head instead of her eyes when tracking a toy; in clip 2, with her head restrained, she struggles to track it. Her mild symmetrical ptosis is also visible.Download Supplementary Video 6 via http://dx.doi.org/10.1212/207241_Video_6

### Molecular Genetic Investigations

Sanger confirmation for the variants in families 1 and 3 is shown in Figure 1. In family 1, 14 rare homozygous variants predicted to change the amino acid sequence or occurring within 10 bases of a splice site were identified, but of these, 10 were consistently predicted by in silico tools to be benign, including Combined Annotation Dependent Depletion scores <20. Of the remaining 4, 2 were associated with clearly irrelevant phenotypes: an autosomal dominant craniofacial disorder in the case of *FGFR1* and an autosomal dominant hepatic disorder for *SEC63*. The *SEC63* variant, moreover, although close to a splice site, was not predicted to have a significant effect on splicing. A third variant, in *IARS2*, was found not to segregate with disease when the similarly affected sibling was tested. This left only a variant in *SLC30A9*: NM_006345.3:c.1253G>T, p.Gly418Val. In silico predictors of pathogenicity consistently predicted a deleterious effect on protein function (Table 1). Heterozygous and compound heterozygous variants in F1(II-2)'s genome were also checked using the same large gene panel, but no additional likely pathogenic candidates were identified. The same variant was subsequently identified in family 2.

**Table 1:**
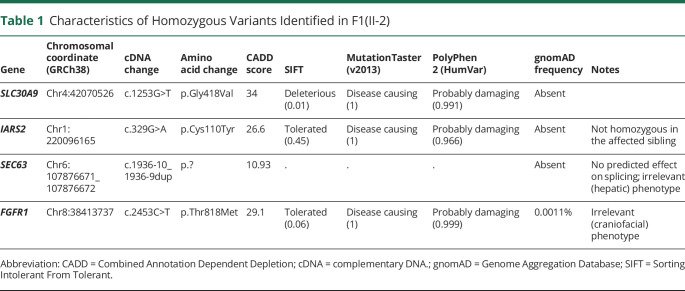
Characteristics of Homozygous Variants Identified in F1(II-2)

For family 3, the previously published pathogenic variant in *SLC30A9* (NM_006345.3:c.1049delCAG, pAla350del) was identified in a region of homozygosity shared between all 4 affected siblings. No other known pathogenic variants in relevant genes were found in these regions. Sanger sequencing confirmed that the variant segregated as expected with disease in the family, with both parents being heterozygous carriers.

In proband 4, compound heterozygous variants in trans were present as follows: NM_006345.3:c.1083dup, p.Val362Cysfs*5 (maternal) and NM_006345.3:c.1413A>G, p.Ser471= (paternal). No other variants likely to be pathogenic were identified. The first variant, as a frameshift, was predicted to result in protein truncation and probably nonsense-mediated decay. The second, synonymous, variant was predicted to affect splicing. As in silico splicing predictors were equivocal, this was verified experimentally (see below).

### Modeling

**Figure 3:**
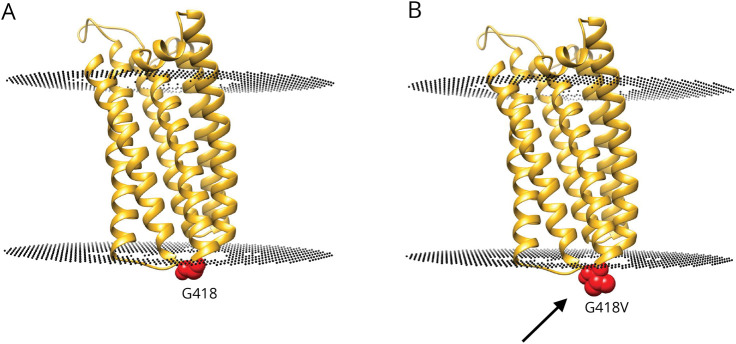
Structure Model of the Transmembrane Domain of Zn-T9 From the AlphaFold^19^ Protein Structure Database (Residues 221–449) The black discs indicate the predicted position of the membrane. (A) Gly418 is shown as red spheres and is present in the loop connecting 2 transmembrane helices. (B) Gly418Val substitution is shown is red on the structural model and indicated with an arrow: valine is predicted to provide less conformational flexibility than glycine, and the substitution is predicted to decrease the stability of the loop and affect transmembrane helix packing.

The novel variant identified in families 1 and 2, p.Gly418Val, is present in the loop connecting 2 transmembrane helices (TM5 and TM6, Figure 3) and is a partially conserved residue, as observed from the multiple sequence alignment obtained using ConSurf. Because of glycine's unique nature, it is known to provide much more conformational flexibility than other amino acids. Glycine, when present in the transmembrane regions, is also reported to facilitate helix packing in membrane proteins and is therefore important for the association of transmembrane helices.^26,27^ Substitution to valine, which has a larger hydrophobic side chain, will decrease the flexibility of the loop and may affect transmembrane helix packing.

### Splicing Experiment

**Figure 4:**
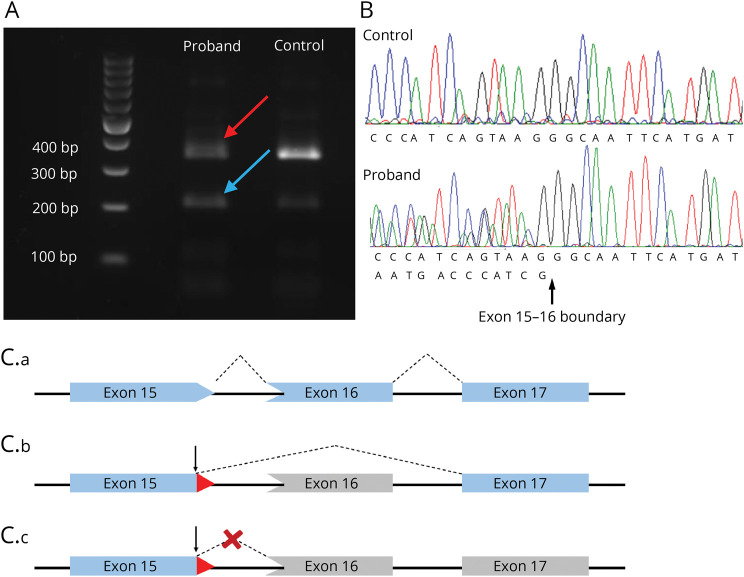
Results of Splicing Assay for the Synonymous Variant Found in F4 (A) Gel electrophoresis of the cDNA from patient F4. Bands on the far left represent the DNA ladder. For both proband and control, 2 bands are present, (red and blue arrows) indicating (at least) 2 splicing products. (B) Chromatogram showing Sanger sequencing results of cDNA isolated from the upper band (red arrow in A). The control shows a single trace; the proband has a double trace commencing at the exon 15–16 boundary indicating the presence of 2 similarly sized splicing products. One of these is wild type; the other (letters below) is missing the final 5 base pairs from exon 15. (C) Schematic showing the different splicing products: (C.a) is the normal larger product, including all of exons 15, 16, and 17; (C.b) is the normal smaller product, in which exon 16 and the final 5 bp of exon 15 are skipped; (C.c) is the abnormal larger product in which the final 5 bp of exon 15 only are skipped, resulting in a frameshift. bp = base pair; cDNA = complementary DNA.

The synonymous variant (c.1413A>G, p.Ser471=) occurs 5 base pairs before the end of exon 15 (in transcript NM_006345.3). Unexpectedly, electrophoresis of fibroblast-derived cDNA from both the proband and 2 unaffected controls revealed a double band, indicating 1 splicing product of the predicted size and a second, smaller product in which exon 16 and the last 5 base pairs of exon 15 were skipped. This suggests that an undocumented additional transcript exists in which the variant is exactly on the exon boundary. Sequencing of the larger band identified that in fact, 2 different products were present in the proband (but not the controls), with a double trace beginning at the site of the variant (Figure 4). These 2 products were too similar in size to be resolved by gel electrophoresis, differing only by 5 bp, but sequencing indicated that the trace unique to the proband had lost the final 5 bp of exon 15. This would be expected to result in a frameshift and premature termination as follows: p.Val472Glyfs*4.

## Discussion

We have described 4 new families (8 affected individuals) harboring different biallelic variants in *SLC30A9*, presenting with a phenotype compatible with BLPS. Our report further enhances knowledge of the phenotypic spectrum of this newly described neurogenetic disorder.

The index kindred, comprising 6 affected individuals, all presented with progressive neurologic deterioration with onset in the first decade of life, after a period of normal early development in infancy. All experienced psychomotor regression or stagnation with dystonia and/or choreoathetosis involving the limbs, camptocormia, and oculomotor apraxia. At least 4 had ptosis, and at least 3 had strabismus. Four of the 6 had hyperechogenic kidneys and hypertension, with evidence of moderate renal dysfunction, and tubulointerstitial nephritis was histologically demonstrated in 1. At the time of their last reported assessment, all were still living, aged between 6 and 19 years.^2^

The single affected individual recently reported by Kleyner et al. had compound heterozygous protein-truncating variants (c.40delA, p.Ser14Alafs*28 and c.86_87dupCC, p.Cys30Profs*30). She presented in infancy with low birth weight, global developmental delay, bilateral sensorineural hearing loss, and unexplained impairment of renal function. Upper limb dystonia presented by around age 5 years and progressed to become generalized. Oculomotor apraxia and ptosis, however, were not seen.^3^

The affected individuals in the additional families we report share the distinctive features of a progressive dyskinetic/dystonic movement disorder, intellectual disability, oculomotor apraxia, and ptosis. Only 1 of our probands (F2) has definite renal dysfunction, although 1 member of family F1 is also known to renal services, and none currently have hypertension or hyperkalemia. Hearing impairment was not found in any of Perez et al.'s index kindred^2^ but was found in the Kleyner et al. proband,^3^ and in at least 3 of the individuals we report here (F1(II-1), F2, and F4): family 3 has not had a detailed audiologic assessment.

*SLC30A9* encodes ZnT-9, a ubiquitously expressed transmembrane protein, which is known to play a role as a zinc transporter. Perez et al., in the same study in which they report on the disease, offer immunofluorescence-based evidence that the protein colocalizes with cytosolic vesicles and especially the endoplasmic reticulum. In vitro, cells expressing the in-frame deletion variant have been found to have lower levels of cytosolic zinc, but disturbances of zinc metabolism in vivo have not yet been demonstrated, and notably, in those of our patients where testing was possible, serum zinc levels were normal. Of interest, people with BLPS do not manifest many of the classical signs of systemic zinc deficiency, such as dermatitis and diarrhea.^28^ This could reflect the fact that a transporter defect would only deplete intracellular, rather than total body, zinc, the consequences of which are not known. It is also possible that ZnT-9 has more physiologic functions than have yet been identified. ZnT-9 is designated as a zinc transporter partly due to homology with other related proteins, but the possibility that it also plays a role in the transport of other metal ions, as is the case for other SLC30 transporters, has not yet been fully explored.^29^

The reported *SLC30A9* protein–truncating variants are likely to mediate a loss-of-function effect through nonsense-mediated decay. Furthermore, both the initially reported in-frame deletion^2^ and the novel missense variant reported here are also likely to exert a loss-of-function effect as they are predicted to affect the cation efflux domain of the protein and will potentially disrupt the structure of its transmembrane helices. We hypothesize that this would impair the regulation of zinc transport (and any other ions for which the protein may act as a transporter), leading to disturbances of metal ion homeostasis at the cellular or subcellular level.

We note that childhood-onset renal impairment does not appear to be an essential feature of BLPS and has only been identified in 2 of our probands (F1(II-2) and F2). Sensorineural hearing impairment has now been reported in individuals from 4 affected families—our families 1, 2, and 4, as well as the individual described by Kleyner et al.^3^ (Table 2).

**Table 2:**
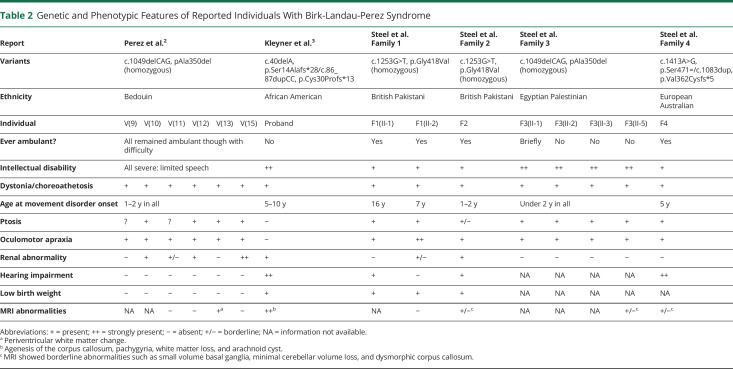
Genetic and Phenotypic Features of Reported Individuals With Birk-Landau-Perez Syndrome

To date, given the relative paucity of reported cases, it is not possible to extrapolate strong genotype-phenotype correlations. In our study, families 1 and 2, harboring a missense variant, have a relatively milder phenotype and better neurodevelopmental outcomes with retained speech and ambulation; the 2 known families who share an in-frame deletion (Perez et al.'s index kindred and our family 3) have an intermediate phenotype with ambulation sometimes gained but then lost and severe intellectual disability; and the individual reported by Kleyner et al., who has biallelic protein-truncating variants early in the protein, has severe intellectual disability and never walked. However, our proband F4, who has biallelic truncating variants (1 due to a splicing defect) much closer to the C-terminus of the protein, has a milder phenotype comparable to our families 1 and 2. Identification of further cases of BLPS will help determine whether specific genetic variants are predictors of phenotypic severity.

In conclusion, BLPS is a newly recognized cause of complex early-onset hyperkinetic movement disorders. Clinicians should consider testing for *SLC30A9* variants in children with undiagnosed progressive dystonic or dyskinetic movement disorders, especially when accompanied by learning disability and oculomotor abnormalities, and particularly in families where a recessive mode of inheritance is suspected. For the time being, we would advocate symptomatic treatment of the movement disorder and regular surveillance of renal function and blood pressure. Future research should focus on further elucidating the underlying pathogenic mechanisms by which *SLC30A9* dysfunction brings about the manifestations of BLPS and how these might be modified through better-targeted precision medicines to improve patient outcomes.

## Supplementary Material

Download Supplementary Video 1

Download Supplementary Video 2

Download Supplementary Video 1

Download Supplementary Video 4

Download Supplementary Video 5

Download Supplementary Video 6

## Glossary

BLPS: Birk-Landau-Perez syndrome
bp: base pair
cDNA: complementary DNA
SNV: single nucleotide variation
WES: whole-exome sequencing
WGS: whole-genome sequencing
